# Comparative Accuracy of Electronic Apex Locators and Conventional Radiography for Working Length Determination in Permanent Teeth: A Systematic Review

**DOI:** 10.7759/cureus.93997

**Published:** 2025-10-07

**Authors:** Naser A Alarifi, Abdulrahman M Alqahtani, Abrar Z Alharbi, Musaed H Alanazi, Abdulrahman M Alanazi, Ahmed F Alharbi, Naif F Alanazi, Khaled M Aloatibi, Fawaz S Alatallah, Haider A Alali, Abdulelah M Koaban

**Affiliations:** 1 Dental Department, Albaha University, Albaha, SAU; 2 General Dentistry, Ministry of Health, Riyadh, SAU; 3 General Dentistry, Ministry of Health, Jeddah, SAU; 4 College of Dentistry, Majmaah University, Majmaah, SAU; 5 General Dentistry, Ram Clinics, Dammam, SAU; 6 Dentistry, King Fahad Medical City, Riyadh, SAU

**Keywords:** electronic apex locator (eal), endodontic treatment outcomes, radiographic accuracy, root canal measurement, working length determination

## Abstract

Accurate working length determination is essential for the success of root canal treatment. While radiographs have traditionally been used for this purpose, electronic apex locators (EALs) have been introduced as an alternative diagnostic tool. This systematic review and meta-analysis compared the accuracy of EALs with conventional and digital radiographs for working length determination in vivo and ex vivo studies. A comprehensive search of PubMed, Cochrane Library, LILACS, EBSCO, and Google Scholar was conducted, and 16 eligible studies were included. Across randomized controlled trials, EALs consistently achieved higher proportions of acceptable working length determinations, ranging from 87.0% to 92.1%, compared with 74.0% to 83.1% for radiographs, while the incidence of overextended cases was markedly lower in EAL groups. In vivo and ex vivo investigations confirmed that EALs provided superior or comparable accuracy to radiographs within the accepted tolerance range of ±0.5-1.0 mm of the apical constriction. Risk-of-bias assessment using QUADAS-2 indicated concerns in patient selection and reference standard domains, although the index test domain was generally low risk. Overall, the evidence suggests that EALs are more accurate and reliable than radiographs in determining working length, supporting their integration into endodontic practice, although further standardized, multicenter studies are needed to reinforce clinical recommendations.

## Introduction and background

Endodontic therapy, or a root canal treatment, aims to eliminate intracanal microorganisms and prevent reinfection by cleaning, shaping, and sealing the root canal system. However, even well-performed cases can fail primarily because of persistent or secondary intraradicular infection [1]. Beyond technical execution, biological control is the crucial factor that influences the outcomes of the procedure [1]. In practice, success also hinges on a precise working length (WL) because WL governs the extent of chemomechanical preparation and the apical seal during obturation [2]. Therefore, precise WL determination is a must for efficient shaping, irrigation, and three-dimensional obturation [2].

Traditionally, WL determination is done radiographically, and radiographs remain essential to assess root and canal morphology and to document treatment progress. However, depending on a single radiographic endpoint is risky as the outcomes may be influenced by apical anatomic variations [3-7]. Classic anatomic studies have shown that the major foramen frequently does not coincide with the radiographic/anatomic apex: across tooth types, approximately 92.4% of major foramina deviated from the anatomic apex, with mean apex-foramen distances commonly around 0.59 mm; buccal/lingual exits in particular cannot be reliably identified on periapical images [3]. Due to these variations, fillings that may seem radiographically ideal may actually be over- or under-extended in reality [3,7]. Age and tooth type further influence apical anatomy. Histologic landmarks such as the cementodentinal junction (apical constriction) lie coronally to the foramen and cannot be identified on periapical images, reinforcing the limits of radiography alone [4,7]. Notably, younger patients have a foramen-to-constriction distance of ≈0.5 mm on average, and older patients have ≈0.8 mm. Posterior teeth also tend to have larger apex-to-foramen distances than anterior teeth [4,7].

To overcome these issues, electrical methods were initially explored. Custer first detected the passage of an instrument through the apical foramen using an electrical approach [5]. Building on this concept, Sunada achieved an electronic WL determination technique reliable to typically within ±0.5 mm; this was done by showing that a consistent circuit reading of about 40 µA (≈6.5 kΩ) occurs when the instrument tip reaches periodontal tissues [6]. The physiologic basis for in vivo apex location done electrically is derived from these studies [6].

Contemporary electronic apex locators (EALs) have evolved from simple resistance meters to sophisticated multi-frequency impedance devices. With these devices, fewer radiographs are required. They can indicate apical foramens that lie away from the radiographic apex, help detect perforations, and reduce the ambiguity of radiographic methods [7]. The best approach remains a combination of techniques, where EALs are used to target the apical endpoint and radiographs for morphology, anomalies, and documentation. In addition, despite the high levels of accuracy offered by EALs near the apical constriction, these devices complement rather than replace thorough imaging [7].

This systematic review evaluates the comparative accuracy of electronic (digital) apex locators versus conventional radiographic methods for WL determination in fully developed permanent teeth. It analyzes evidence from randomized clinical trials, in vivo clinical studies, and ex vivo experiments. In addition to pooled accuracy within clinically meaningful thresholds (±0.5 mm and, where available, ±1.0 mm), the review also examines tendencies to over- or underestimate sources of heterogeneity related to tooth type and canal conditions, reference standards, and device generation. Finally, it discusses the practical implications for minimizing radiographic exposure while maintaining robust documentation.

## Review

Rationale

Although systematic reviews on EALs exist, most have not collectively evaluated evidence across randomized controlled trials, in vivo studies, and ex vivo experiments. A comprehensive synthesis is therefore warranted to elucidate the accuracy and reliability of EALs compared with conventional methods in permanent teeth.

Objectives

This systematic review aims to evaluate the accuracy of digital apex locators compared to conventional radiographic methods for the measurement of WL in permanent teeth.

Methods

The present systematic review is reported according to the Preferred Reporting Items for Systematic Reviews and Meta-Analyses (PRISMA) statement [8].

Eligibility Criteria

As per the PICO (Patient/Population, Interventions, Comparison, Outcomes) framework, studies conducted on fully developed human permanent teeth with closed apices, either in vitro or ex vivo (extracted), were included. The intervention involved EALs or endo motors with integrated apex locators, while the comparisons were made with conventional radiography, i.e., radiovisiography (RVG) or intraoral periapical radiographs (IOPA) to evaluate WL. The eligible studies comprised randomized controlled trials, non-randomized controlled trials, in vitro studies, ex vivo studies, and diagnostic comparative studies. The primary outcomes were (i) adequacy of obturation in randomized controlled trials with control groups, assessed on final WL radiographs, and (ii) accuracy of WL determination in in vivo and ex vivo diagnostic studies, defined as measurements within ±0.5-1.0 mm of the apical constriction or as mean error. Secondary outcomes included proportions of short or overextended working lengths, postoperative pain, and radiation exposure, when reported.

Studies performed on primary teeth, artificial teeth, teeth with open apex, resorption, calcification, or perforation, as well as previously endodontically treated teeth, were considered ineligible. Studies involving teeth with unusual root canal morphology were excluded to avoid anatomical variations from affecting WL accuracy. Research without valid and clearly defined comparison groups was excluded. Studies that compared methods without a reference standard (actual WL) were excluded. Studies conducted in vitro or ex vivo with a sample size below 50 teeth were excluded to ensure adequate statistical power and reliability of results. Furthermore, case reports, case series, reviews, finite element analyses, book chapters, expert opinions, animal studies, and incomplete studies were omitted.

For consistency, studies were categorized according to the setting in which WL determination was performed. In vivo studies were those conducted clinically on teeth in the oral cavity prior to extraction. Ex vivo studies were those conducted on extracted human teeth under laboratory or simulated conditions (e.g., embedded in alginate or resin), even if the authors described them as in vitro. In vitro was reserved only for studies performed on artificial or synthetic models such as resin blocks or plastic teeth. Any mislabeling by the studies’ original authors was corrected in this review to maintain consistency. Table 1 summarizes the PICOS framework used to define study eligibility.

**Table 1:** PICOS framework for eligibility of included studies PICOS: Patient/Population, Interventions, Comparison, Outcomes

Element	Description
Population (P)	Human permanent teeth with closed apices (in vivo prior to extraction or ex vivo on extracted teeth); studies restricted mostly to single-rooted teeth.
Intervention (I)	Electronic apex locators (EALs) or endomotors with integrated apex locators.
Comparator (C)	Conventional radiography (intraoral periapical radiograph [IOPA] or radiovisiography [RVG]); some studies also included the tactile method.
Outcomes (O)	Primary: (a) Adequacy of obturation (in RCTs with control groups, using final WL radiographs); (b) Accuracy of working length determination (in vivo and ex vivo diagnostic studies, within ±0.5–1.0 mm of the apical constriction or as mean error in mm). Secondary: Proportions of short/overextended WL, postoperative pain, and radiation exposure (where reported).
Study design (S)	Randomized controlled trials, non-randomized comparative in vivo studies, and ex vivo diagnostic accuracy studies on extracted human teeth.

Information Sources and Search Strategy

A systematic search was conducted across PubMed, Cochrane Library, EBSCOhost, LILACS, and Google Scholar to identify relevant studies. The search included a combination of MeSH terms and free-text keywords related to apex locators, periapical radiography, dental pulp, and tooth apex. The last search was performed on August 13, 2025, for each database. Additionally, the reference lists of all included articles and relevant reviews were manually screened to identify any studies that may have been missed in the electronic search. No trial registers or professional organization websites were searched separately. Only studies published in English and involving human participants were considered. The detailed search strings used for each database are provided in Table 2.

**Table 2:** Database-specific search strategies

Database	Search String	Filters Applied	Hits Retrieved
PubMed	("Apex Locator" OR "Electronic Apex Locator" OR EAL)) AND ("Radiography, Dental/methods"[Mesh] OR periapical radiograph* OR digital radiograph*) AND (("Dental Pulp Cavity"[Mesh] OR "Tooth Apex"[Mesh])	Full-text; English; Humans	1051
LILACS	(apex locator*) AND (radiograph*) OR (radiografia periapical)	English only	106
Cochrane Library	#1 (Apex locator):ti,ab,kw; #2 MeSH descriptor: [Radiography, Dental] explode all trees; #3 MeSH descriptor: [Radiography, Dental, Digital] explode all trees; #4 #1 AND #2 OR #3	English	102
EBSCO	TI ( "apex locator" OR "electronic apex locator" ) AND AB ( "working length" OR "root canal length" OR "endodontic length" OR radiograph* )	English	53
Google Scholar	"electronic apex locator" OR “ROOTZX” AND “periapical radiograph” OR “Conventional radiograph” OR "working length" accuracy AND “permanent teeth”	English; Human; Randomized Controlled Trials; Experimental Studies	533

Selection Process

Two reviewers independently screened titles and abstracts of all retrieved records to identify potentially eligible studies. The full-text articles were again assessed by the same two reviewers independently by using the same eligibility criteria. Rayyan software was used for deduplication and blinded screening of the records. Any disagreements were resolved through discussion until a consensus was reached.

Data Collection Process

Two reviewers independently extracted relevant data from each included full-text article using a standardized data extraction form. The variables collected included author name, publication year, study design, sample size, type of tooth, method of WL determination, use of irrigants, comparator method, outcome definitions, and key numerical results. Rayyan software was used to organize and manage the extracted data. No automation tools were used for data extraction. In case of inconsistencies between the two reviewers, the extracted data were compared and discussed until an agreement was reached. No contact was made with study investigators for missing data.

Data Items

The primary outcome was the accuracy of WL determination, defined as agreement between the EAL and the reference standard. Secondary outcomes included mean measurement error, proportion of values within clinically acceptable tolerance (±0.5-1 mm), and reliability statistics. Additional data items were study characteristics, tooth type, intervention, and comparator details.

Study Risk-of-Bias Assessment

Risk-of-bias was assessed using tools specific to the study design. Four randomized controlled trials (RCTs) were evaluated with the Cochrane RoB 2.0 tool, which examines bias in the randomization process, deviations from intended interventions, missing outcome data, outcome measurement, and selective reporting. Seven in vivo and five ex vivo diagnostic accuracy studies were assessed using the QUADAS-2 tool, which evaluates potential bias in patient selection, index test, reference standard, and flow and timing [9].

Assessments were independently performed by the same two reviewers, and any disagreement was resolved via discussion until a consensus was reached. The risk-of-bias assessment was summarized in both tabular and graphical formats (traffic light).

Effect Measures

The primary effect measure extracted from each study was the mean difference (mm) between the EAL-determined WL and the reference standard (radiograph, stereomicroscope, or direct visualization). Where reported, the proportion of measurements within clinically acceptable limits (±0.5 mm or ±1 mm) was also collected. Additional effect measures included absolute error values, intraclass correlation coefficients, and statistical test results.

Synthesis Methods

Studies were grouped for analysis based on their design (in vivo, ex vivo, or RCT) and the comparator used (EAL vs. radiographic WL determination). Eligibility for synthesis was checked by listing the intervention and comparator details and ensuring they matched the review objectives. Data were then summarized in both narrative form and structured extraction tables. To enable comparison across studies, measurement units and error definitions were standardized. The results were displayed using summary tables and traffic light risk-of-bias figures. No automation tools were used.

Due to differences in study designs, reference standards, and reporting metrics, a meta-analysis could not be performed. Instead, a qualitative synthesis was carried out to identify patterns in measurement accuracy, error ranges, and agreement statistics.

Heterogeneity was addressed descriptively by comparing results across subgroups. Sensitivity analysis was not performed due to insufficient data consistency.

Certainty Assessment

Given the substantial heterogeneity and absence of a meta-analysis, a formal GRADE-based certainty assessment was not feasible. Instead, the strength of evidence is described narratively.

Results

Study Selection

The initial database search produced 1848 records. After deduplication using Rayyan software, 1582 records remained. Title and abstract screening excluded 1394 records, leaving 188 that were retrieved for full-text assessment. During full-text screening, several studies were excluded for not meeting the eligibility criteria. The most common reasons were as follows: (1) use of ineligible populations (immature apices, primary teeth, retreatment cases, teeth with vertical fractures, or unusual root morphologies); (2) inappropriate interventions or comparators (studies evaluating only different EALs without radiographic reference or wrong comparison groups); (3) outcomes not measuring WL accuracy (pain assessment using a visual analog scale); and (4) ineligible study designs (review articles, case reports).

**Figure 1:**
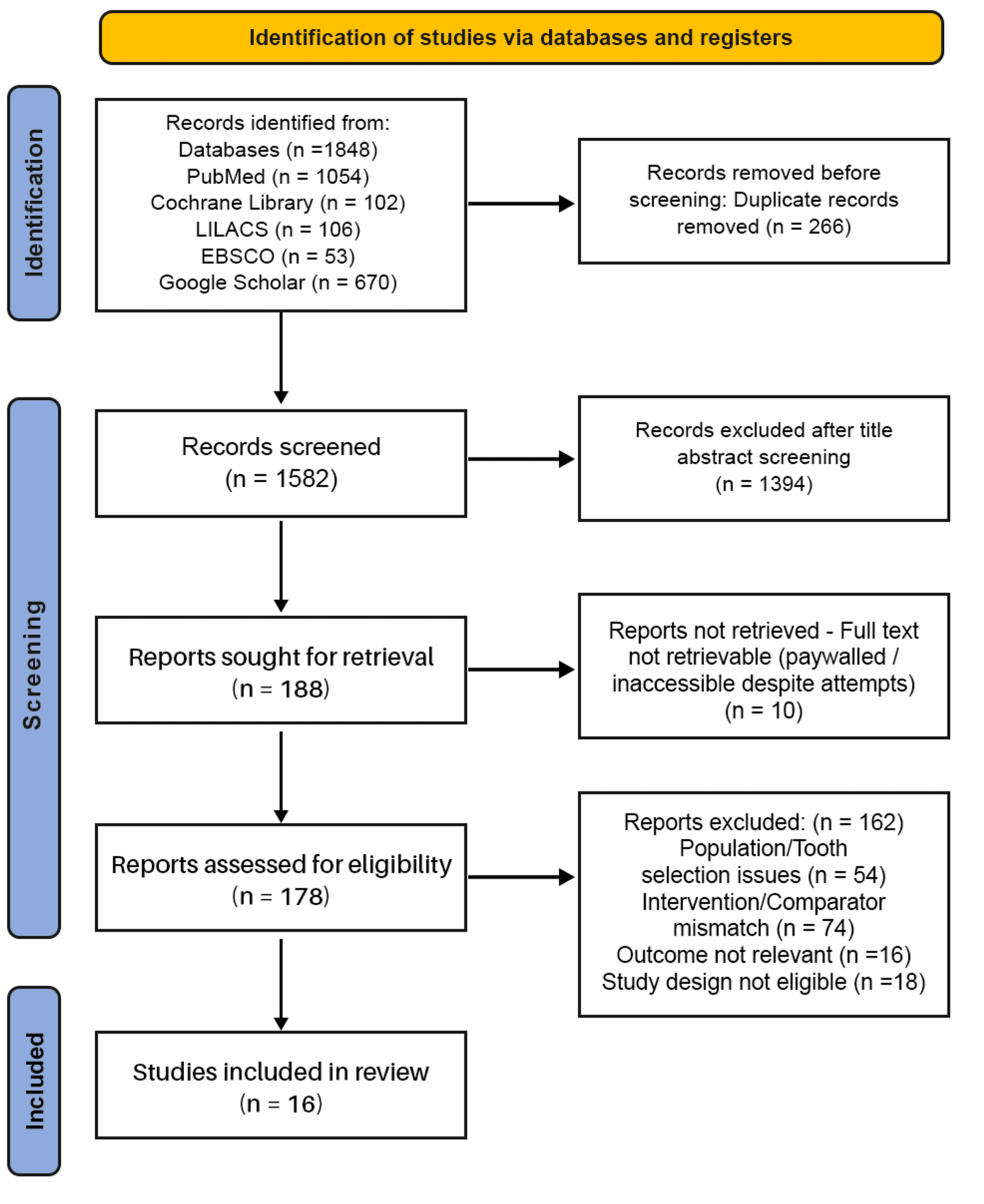
PRISMA Flow Diagram for Study Selection

A total of 10 full-text reports could not be retrieved due to paywall restrictions, despite attempts through institutional access and contacting the study authors. These were documented under "Reports not retrieved” in the PRISMA flow diagram (Figure 1). Ultimately, 16 studies met all inclusion criteria and were included in the qualitative synthesis.

Study Characteristics

The key characteristics of all included studies are summarized in Table 3, including study design, sample details, EAL devices, comparator methods, operator information, and reported outcomes. Among the included studies, four were RCTs, seven were in vivo studies, and five were ex vivo studies. Studies were categorized based on the timing of WL (WL) determination. In vivo studies were defined as those in which WL was measured clinically in the oral cavity during treatment, since the intervention occurred prior to extraction. Typically, in these studies, WL was determined using both EALs and radiographic methods, with confirmation by stereomicroscopy after extraction. Ex vivo studies, conversely, were experiments conducted on extracted teeth, where WL was determined using the test methods, and the reference standard was established under magnification. For consistency, this classification was applied across all included studies. The sample sizes in the included studies ranged from 50 to 245 teeth. Many studies were conducted on single-rooted, single canal teeth, whereas others included a broader variety of tooth types. The apex locators used in the intervention group were RootZX, RootZX mini, Raypex 5, Elements-Diagnostic, Precision AL, Sendoline S5, Apex ID, Endex, ProPex II, DentAport ZX, Woodpex V, and Woodpex III. These apex locators were compared with either conventional periapical radiographs, radiovisiographs (RVG), endomotors with integrated apex locators, or tactile methods.

**Table 3:** Data extraction summary of included studies RCT: randomized controlled trial; NR: not reported; CI: central incisors; LI: lateral incisors; AC: apical constriction; DDR: direct digital radiography; RVG: radiovisiography; EAL: electronic apex locator; IAL: integrated apex locator. “Acceptable cases” refer to working length determinations falling within the accepted tolerance range (±0.5–1.0 mm of the apical constriction) as defined in each study. Reference standards included radiographic evaluation, stereomicroscope examination, or direct apical exit observation, depending on the study design. Accuracy percentages and statistical results are reported exactly as presented by the original authors. The apex locators used in the intervention group included Root ZX and Root ZX mini (J. Morita, Kyoto, Japan), Raypex 5 (VDW, Munich, Germany), Elements-Diagnostic Unit (SybronEndo, Orange, CA, USA), Precision AL (Denjoy, Changsha, China), Sendoline S5 (Sendoline, Täby, Sweden), Apex ID (Kerr Dental, Brea, CA, USA), Endex (Osada, Tokyo, Japan), ProPex II (Dentsply Maillefer, Ballaigues, Switzerland), Dentaport ZX (J. Morita, Kyoto, Japan), Woodpex III and V (Guilin Woodpecker Medical Instrument Co., Guangxi, China), and CanalPro Apex Locator (Coltene/Whaledent, Altstätten, Switzerland).

Sr. No	Author (Year)	Study Design	Sample Size (n)	Tooth Type	Intervention (Index Test) EAL Brand Used	Comparator	Reference Standard	Key Findings/Accuracy	Conclusion
	Singh et al. (2015) [10]	RCT	153 Patients, 153 teeth, 153 canals	Single-rooted teeth	Raypex® 5	Periapical radiograph	Mastercone radiograph evaluation	“Acceptable cases” Radiograph group = 83.1% EAL group = 92.1% “Over cases” Radiograph group = 13.1% EAL group = 2.6%	Significantly lesser over obturated cases in the EAL group (P = 0.017)
	Koçak et al. (2013) [11]	RCT	120 patients, 283 roots	NR	Root ZX mini	1. Conventional Radiograph; 2.Integrated apex locator (VDW Gold)	Mastercone (final) radiograph evaluation	“Acceptable cases” Radiograph group= 81.9% EAL group = 87.0% IAL group= 83.5%	Highest rate of short and over-filling in Radiograph group, but no statistically significant difference between the three groups (P = 0.894)
	Jarad et al. (2011) [12]	RCT	46 teeth	24 single-rooted; 22 multirooted	Raypex® 5	Periapical radiograph	Mastercone radiograph evaluation	“Acceptable cases” Radiograph group= 74% EAL group= 91%	No significant difference observed in both groups at the 5% level
	Ravanshad et al. (2010) [13]	RCT	84 patients, 188 canals	NR	Raypex® 5	Periapical radiograph	Mastercone & final radiograph evaluation	“Acceptable cases” Radiograph group = 85.7% EAL group = 90.4% “Over cases” Radiograph group = 13.1% EAL group = 8.7%	EALs quite comparable if not superior to radiographs but no statistically significant difference. Significantly lower “over” mastercone results in EAL group (P = 0.00)
	Vieyra et al. (2011) [14]	In-vivo study	245 teeth, 693 canals	CI = 17; LI= 26; Canine= 12; Premolar= 28; Molar=162	Root ZX®, Elements-Diagnostic, Precision AL, and Raypex 5	Periapical radiographs	Apical 5 mm sectioned and examined under a stereomicroscope	Measurements 1.0mm through the AC Radiographs = 31.4%. EALs= 0.6% (average)	No significant difference in the accuracy of the four EALS.
	Khan et al. (2021) [15]	In vivo	60 patients, 60 teeth, 60 canals	Premolars	Root ZX®	Digital radiographs	Apical 1/3 sectioned and examined with help of scale and endodontic loupes	Accuracy within 0.5mm of apex EAL = 95% Digital Radiograph = 70%	EAL is more accurate as compared to digital radiography
	Saha et al. (2024) [16]	In-vivo study	50 teeth, 50 canals	Single-rooted teeth	Root ZX mini	Periapical radiograph	Direct apical exit of the file observed using stereomicroscope and subtracting 0.5mm	Gold standard = 20.71 ± 2.12 mm Conventional radiograph = 20.57 ± 2.19 mm / 58% precision (n = 29) EAL = 20.72 ± 2.12 mm / 80% PRECISION (n= 40) with tolerance range off ± 0.5 mm	Root ZX Mini is significantly more accurate compared to radiography (P = 0.004)
	Osei-Bonsu et al. (2023) [17]	In vivo	96 teeth, 96 canals	Single-rooted, single canal teeth	Sendoline S5	1. Tactile sensation; 2. Digital Radiography	Apical 4-5 mm sectioned and examined under a digital microscope	Accuracy in EAL = 96.9%, 31 teeth Digital radiograph = 59.4%, 19 teeth Tactile sensation = 25%, 8 teeth	EAL provided more reliable and accurate results compared to digital radiography and tactile methods (P = <0.001)
9	Vieyra et al. (2018) [18]	In vivo	120 patients, 247 canals	Anteriors, Premolars, and Molars	RootZX, Apex ID, Raypex 6, Mini Apex Locator, Canal Pro	Digital Radiography	Apical 5 mm sectioned and examined under stereomicroscope	Anteriors= 88.05%, 83.6%, 83.6%, 80.6% and 52.2%; Premolars = 93.75%, 87.5%, 93.7%, 87.5% and 37.5%; Molars = 83.78%, 81.08%, 63%, 83.78%, 78.37% and 21.62%	No statistically significant difference between the four EALs; a significant difference between the EALs and radiographs. (P = 0.05)
10.	Ullah et al. (2017) [19]	In vivo	50 teeth, 50 canals	Anterior and Premolars	Elements-diagnostic Unit	1. Conventional radiograph; 2. Direct digital radiography	Apical 1/3 sectioned and examined under ×4 magnification	At Apical Constriction, EAL = 54%, Conventional = 40%, DDR = 38 %;Within ±0.5mm EAL = 96%, Conventional = 72, DDR = 76%; Within ±1mm - All three 100%.	No statistically significant difference between EAL & Actual working length (P = 0.345), significant difference between EAL & Conventional /DDR(P <0.001).
11	Zand et al. (2011) [20]	In vivo	75 teeth, 75 canals	Maxillary central and lateral incisors	Root ZX®	Periapical Radiograph	Direct apical exit of the file observed using stereomicroscope and subtracting 1 mm	Descriptive evaluation: 72% (n = 54) of the specimens, both methods had errors in the same direction, and in 28% (n = 21) of the specimens, the two methods had errors in opposite directions.	No significant differences between radiography, Root ZX, and direct visualization. (α = 0.986, p < 0.001)
12.	Cianconi et al. (2010) [21]	Ex vivo	101 teeth	NR	Endex, ProPex II & Root ZX®	Radiovisiography (RVG)	Direct apical exit of the file observed using stereomicroscope with 5× Magnification	The apical foramen (± 0.5 mm) 84.1%, 62.4%, and 82.2%; tendency toward overestimation: 15.9%, 37.6%, and 17.8%, for Endex, Root ZX, and ProPex II, respectively.	Endex and ProPex II were more accurate than Root ZX (p<0.001). EALs are more accurate than RVG.
13.	Saeed et al. (2011) [22]	Ex vivo	50 teeth, 50 canals	Single-rooted teeth	DentAport ZX	1. Conventional Radiography; 2. Digital Radiography	Direct apical exit of the file observed	Failure rate: Conventional = 38%, Digital Radiography = 30%, EAL = 22%; Accuracy of Conventional + EAL = 90%, Digital Radiography + EAL = 96%.	A combination of digital radiographic and apex locator methods considered safe, reliable and precise.
14	Ramezani et al. (2022) [23]	Ex vivo	58 teeth, 58 canals	Maxillary premolars: one single canal or two canals (Vertucci’s type II with one apical foramen)	Root ZX, Woodpex V, and Woodpex III	Digital radiography	Direct apical exit of the file observed using stereomicroscope	Accuracy within ±0.5 mm from the apical constriction = 87.93%, 89.66%, 100%, and 84.48%; Within ±1 mm = 100%, 100%, 100%, and for Woodpex III, Woodpex V, Root ZX, and 96.55% digital radiography, respectively	All the tested modalities showed acceptable accuracy for WL determination in maxillary premolars.
15.	Mancini et al. (2011) [24]	Ex vivo	80 teeth, 120 canals	Molars=40; Bicuspids = 40; Anterior = 40	Endex, RootZX, ProPexII	Radiovisiography (RVG)	Direct apical exit of the file observed using stereomicroscope with 5× magnification	Apical foramen (±0.5mm) for Endex, RootZX, and ProPexII: (1) in anterior teeth, 80.8%, 61.5%, and 76.9%, respectively; (2) in bicuspids, 93.1%, 69%, and 89.7%, respectively; and (3) in molars, 80.4%, 58.7%, and 82.6%, respectively.	EALs more accurate in detecting the apical foramen in bicuspids than in molars and anterior teeth; Radiographic measurements not reliable for determining WL in all dental groups.
16.	Singh AK et al (2021) [25]	Ex vivo study	80 teeth, 80 canals	Single canal Premolars	Root ZX mini	1. Tactile method; 2. Digital radiographs; 3. Endodontic motor with integrated apex locator	Direct apical exit of the file observed and measured with endodontic ruler	Actual WL= (21.84 ± 0.486); EAL= (21.56 ± 0.862); IAL= (20.96 ± 1.010); Digital radiography= (20.74 ± 1.030); Tactile method= (20.42 ± 1.002)	EAL values were nearer to the actual WL as compared to endodontic motors with incorporated apex locator, digital radiograph, and tactile techniques, but not statistically significant. (P = 0.648)

The apex locators used in the intervention group included Root ZX and Root ZX mini (J. Morita, Kyoto, Japan), Raypex 5 (VDW, Munich, Germany), Elements-Diagnostic Unit (SybronEndo, Orange, CA, USA), Precision AL (Denjoy, Changsha, China), Sendoline S5 (Sendoline, Täby, Sweden), Apex ID (Kerr Dental, Brea, CA, USA), Endex (Osada, Tokyo, Japan), ProPex II (Dentsply Maillefer, Ballaigues, Switzerland), Dentaport ZX (J. Morita, Kyoto, Japan), Woodpex III and V (Guilin Woodpecker Medical Instrument Co., Guangxi, China), and CanalPro Apex Locator (Coltene/Whaledent, Altstätten, Switzerland).

Risk of bias in studies

**Figure 2:**
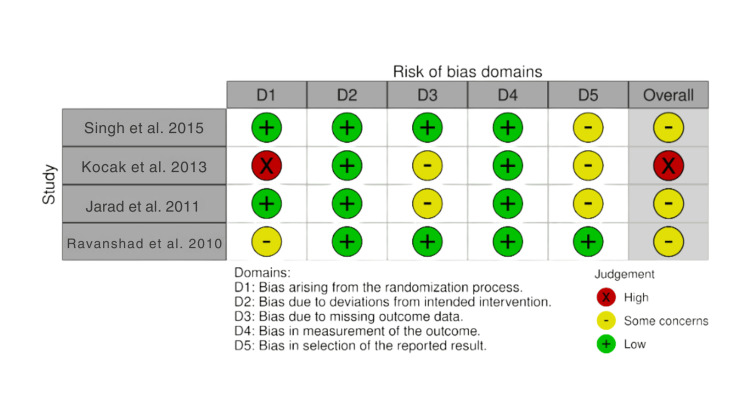
Risk-of-bias traffic light plot for randomized controlled trials (RoB 2) Risk-of-bias assessment conducted using the Cochrane Risk of Bias 2.0 (RoB 2)

**Figure 3:**
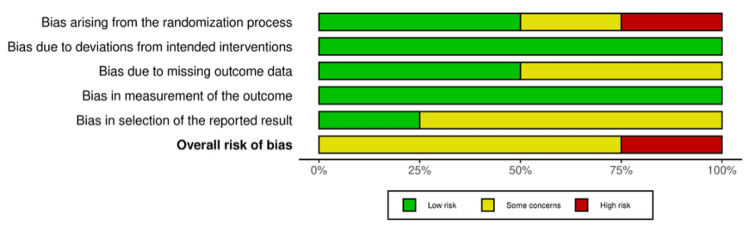
Risk-of-bias summary plot for randomized controlled trials (RoB 2) Each bar represents the proportion of studies judged as low risk (green), some concerns (yellow), or high risk (red) within each domain and for the overall risk of bias.

Three RCTs showed some concerns of bias, and one showed a high risk of bias. In three studies, the domain of selective reporting (bias in selecting the reported results) was judged as “some concerns” in line with RoB 2 guidance due to the absence of a registered protocol [10-12]. Rivanshad et al. presented some concerns, as the randomization was carried out by coin flipping without allocation concealment, although blinding of outcome assessment reduced the risk in other domains [13]. Koçak et al. reported randomization but did not describe the method in detail, and there were additional concerns regarding blinding procedures in this study [11]. As Jarad et al. reported some dropouts, the risk of bias due to missing outcome data was judged as some concerns [12]. The traffic light plot and summary plot for the RoB 2 assessment are presented in Figures 2-3.

**Figure 4:**
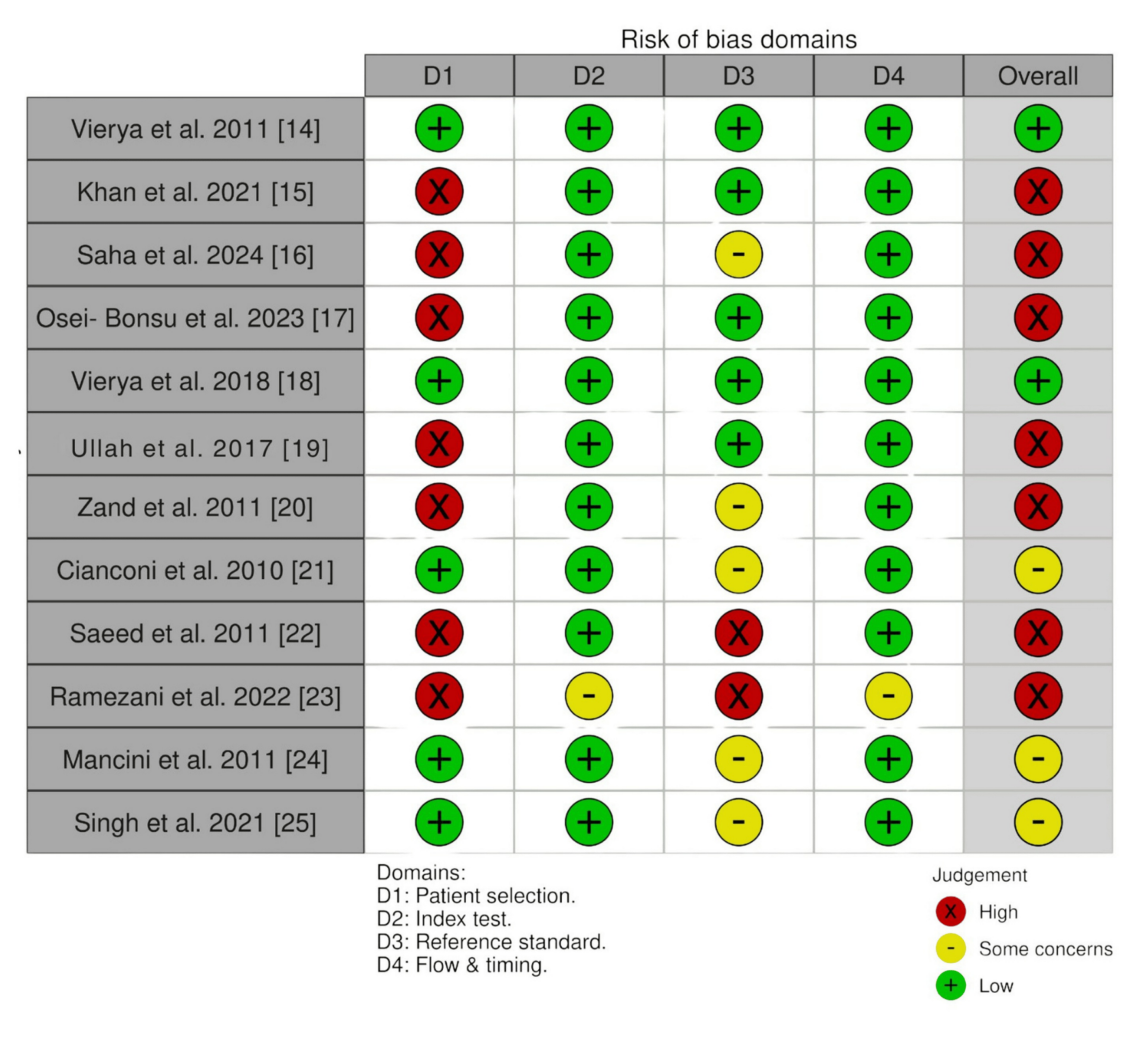
Risk-of-bias traffic light plot for in vivo and ex vivo studies assessed using the QUADAS-2 tool.

**Figure 5:**
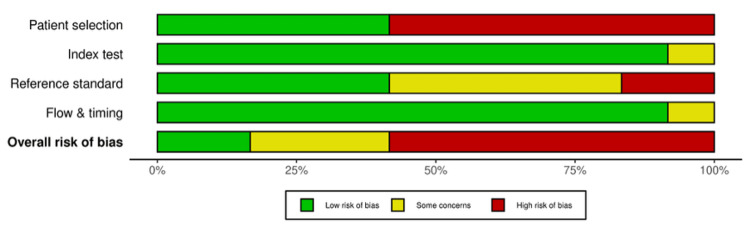
Risk-of-bias summary plot for in vivo and ex vivo studies, assessed using the QUADAS-2 tool. Each bar represents the proportion of studies judged as low risk (green), some concerns (yellow), or high risk (red) within each domain and for the overall risk of bias.

Figures 4-5 present the risk-of-bias assessment for the included in vivo and ex vivo diagnostic accuracy studies, evaluated using the QUADAS-2 tool. The traffic light plot (Figure 4) displays individual study judgments across each domain, while the summary plot (Figure 5) illustrates the proportion of studies rated as low risk, high risk, or with some concerns. Together, these figures provide a visual overview of the methodological quality and potential biases that may influence the interpretation of findings.

Risk-of-bias assessment using QUADAS-2 showed that most studies were at high risk overall (Figures 4-5). Randomization was not applicable in the included in vivo and ex vivo studies because the same teeth underwent all index tests and the reference standard. Furthermore, most studies restricted samples to single-rooted teeth, which may not represent the clinical spectrum. This introduces potential bias, particularly in patient selection [15-17,19,20,22,23,25]. The index test domain was generally judged as low risk, since the EAL measurements were performed without prior knowledge of the reference standard, except in the study by Ramezani et al. [23], in which it was performed prior to the index test [23]. The reference standard domain showed mixed results, with some studies judged as high risk due to reliance on a fixed 0.5 mm subtraction from the apical foramen to define the actual WL [16,20-25]. In one study, the timing of reference standard measurement relative to extraction was not reported [23]. As it was unclear whether the interval was immediate or delayed, this domain was judged as having some concerns. Statistical assessment of publication bias was not applicable due to heterogeneity in study designs (RCTs, in vivo studies, and ex vivo studies).

Results of individual studies

Across all four RCTs, the proportion of acceptable cases was consistently higher in the EAL groups compared with the radiograph groups. Reported acceptable rates in the EAL groups were 92.1%, 87.0%, 91%, and 90.4%, compared with 83.1%, 81.9%, 74%, and 82.1%, respectively, in the radiograph groups [10-13]. Singh et al. and Ravanshad et al. further noted that radiographs tended to overestimate WL, with 13.15% and 10.7% of cases, respectively, extending beyond the apical limit, whereas this was significantly reduced in the EAL groups (2.63% and 1.0%, respectively) [10,13]. Koçak et al. reported that even though endomotor-integrated apex locators yielded a comparable number of acceptable cases (83.5%), they were not superior to EALs, and the difference was not statistically significant (p = 0.0894) [11]. Jarad et al. found that the mean distance from the radiographic apex in the EAL group was 1.06 mm, compared with 1.23 mm in the radiograph group, a difference that was not statistically significant [12]. Overall, although not all comparisons reached statistical significance, EALs demonstrated consistently superior performance across several studies. Table 4 provides a summary of these comparisons, showing that EALs achieved more acceptable cases and fewer overextended results than radiographs.

**Table 4:** Proportion of acceptable, short, and overextended working length determinations in randomized controlled trials. Comparison of working length determinations between electronic apex locators and radiographs in four randomized controlled trials. “Acceptable” = measurements within the accepted tolerance (±0.5–1.0 mm of the apical constriction), “Short” = measurements ending before this range, and “Over” = measurements extending beyond the apical limit. Values are reported as percentages from the original studies.

Studies	EAL			Radiograph		
	Acceptable	Short	Over	Acceptable	Short	Over
Singh et al. [10]	92.10%	5.26%	2.63%	83.1%	3.947%	13.15%
Kocak et al. [11]	87.0%	4.3%	8.7%	81.9%	7.4%	10.6%
Jarad et al. [12]	91%	9%		74%	26%	
Rivanshad et al. [13]	90.4%	8.7%	1.0%	82.1%	7.1%	10.7%

Seven in vivo studies compared (EALs) with radiographic methods for WL determination. Across all, EALs demonstrated higher accuracy and fewer errors. One study by Vieyra et al. reported accuracies up to 89% with EALs versus 15-33% with radiographs, with radiographs showing more frequent overestimations (20-47%) [14]. Similar trends were observed by Khan et al. and Osei-Bonsu et al., who reported EAL accuracies of 95-97% compared with 59-70% for radiographs; tactile methods performed the worst at 25% [15,17]. Saha et al. found that the Root ZX Mini significantly reduced error, with 80% accuracy at ±0.5 mm and 100% at ±1 mm, compared with 58% and 80% for radiographs [16]. Furthermore, Vieyra et al. confirmed that different EALs performed similarly and were all more accurate than radiographs (22% accuracy, 35% overestimations) [14]. Ullah et al. reported 96% accuracy within ±0.5 mm for EALs versus 72-76% for radiographs, with all methods accurate within ±1 mm [19]. Only Zand et al. found no significant differences, though descriptive data still showed fewer clinically relevant deviations with EALs [20].

Four ex vivo studies evaluated EALs against radiographic techniques. Cianconi et al. and Mancini et al. tested Endex, Root ZX, and ProPex II. They found higher accuracy at the apical constriction with EALs (often ≥ 80% for Endex/ProPex II and up to 93.1% in bicuspids with Endex) than with radiographs (≈38-55% correct tip position by plane) [21,24]. Mancini et al. reported a systematic overestimation with EALs (mean 0.29-0.37 mm) and no underestimation, whereas radiographs tended to underestimate measurements [24]. Cianconi et al. similarly showed mean EAL deviations of 0.23-0.50 mm and overestimation rates of 15.9-37.6%, while radiographic agreement with the apex was 44.5-51.5% with underestimation bias [21]. Saeed et al. observed high agreement of all methods with actual length but lower failure with EALs (22%) than conventional (38%) or digital radiography (actual ≈30%), with combined EAL+radiography improving success to 90-96% [22]. Singh et al. found EALs and an integrated-motor AL produced WLs closest to actual (21.56 ± 0.86 mm; 20.96 ± 1.01 mm) versus radiographic and tactile techniques, though differences were not statistically significant [25]. Overall, ex vivo evidence consistently favored EALs, showing smaller deviations and fewer clinically relevant errors than radiographic or tactile methods.

Results of Syntheses

Across different study types, the direction of effect was consistent: EALs produced higher rates of acceptable WL determinations and smaller measurement errors than radiographic methods. In the four RCTs, acceptable case rates in EAL groups ranged ~87-92% versus ~74-83% for radiographs. Two RCTs, Singh et al. and Ravanshad et al., reported statistically significant reductions in over-obturation with EALs [10,13]. In vivo and ex vivo studies also reported similar patterns: EAL accuracy within ±0.5 mm commonly exceeded 80%, while radiographic accuracy was typically in the 20-75% range depending on tooth type and technique. Differences among EAL models were generally small and not statistically significant.

Altogether, the synthesized results consistently favor the use of EALs over radiography for WL determination. However, the absence of pooled estimates and the presence of study-level risk-of-bias concerns limit the strength of conclusions, and the findings generally warrant cautious interpretation.

Certainty of Evidence

Based on the risk-of-bias assessment and consistency across studies, the certainty of evidence was rated as moderate. Although most studies consistently demonstrated the superiority of EALs over radiographs, the overall certainty was downgraded due to methodological limitations, including ex vivo designs, small sample sizes, and limited blinding.

Discussion

Principal Findings

This review synthesized evidence from RCTs, in vivo studies, and ex vivo experiments comparing EAL with radiographic and tactile methods for WL determination. The findings were consistent and clinically significant across different studies: acceptable WL determinations in RCTs for EALs were ~87-92% versus ~74-83% using radiographs [10-13]. Two trials also demonstrated EALs had significantly fewer overextended fillings (≈1-3%) than radiographs (≈11-13%) [10,13]. Similarly, in vivo studies commonly reported accuracy levels of ≥80-97% within ±0.5 mm for EALs, while radiographic levels of accuracy ranged from ~22-76% with a greater chance of overestimation [14-20]. Ex vivo studies supported these findings, with EALs typically demonstrating ≥80-93% accuracy within ±0.5 mm and frequently outperforming radiography. Additionally, a combination of EAL and radiography yielded the highest success (≈90-96%) [21-23]. Variations between different EAL devices were generally minimal, and most studies found comparable performance among contemporary models [18,23]. However, some ex vivo findings favored Endex/ProPex II over Root ZX [21]. Overall, the findings indicate the accuracy of an EAL-first strategy for WL, which can be supplemented by radiographs for morphology and documentation purposes.

Scope of Included Studies

Only studies involving permanent teeth were included to maintain clinical coherence. Primary teeth were excluded from consideration as physiologic resorption and anatomy can confound WL accuracy [4,7]. As most studies restricted samples to single-rooted teeth and excluded complex morphologies [10,12,15-17,19,20,22,25], the findings may not be applicable to multirooted and anatomically challenging cases. Future research focusing on WL determination in molars and retreatment scenarios is therefore recommended.

Sources of Heterogeneity

The observed variability in the findings is likely due to several study-level factors: (i) selection of teeth, whether single-rooted or molars [14,18,24]; (ii) canal environment and the use of irrigants, as these may influence impedance readings but were inconsistently reported [14-20,21-25]; (iii) direct microscopic visualization vs. fixed subtractions from the foramen as the reference standard, as the latter technique may bias accuracy toward a presupposed target [16,20-25]; (iv) ±0.5 mm vs. ±1.0 mm accuracy thresholds [16,19,23]; and (v) device generation/integration (stand-alone vs. endomotor-integrated), as integrated systems are not confirmed to be better than dedicated EALs [11,25]. Due to these factors, the findings are best interpreted qualitatively.

Clinical Implications

Overall, the findings support the use of EALs as the primary method of measuring WL at or near the apical constriction, in addition to confirmatory radiographs for documentation, complex anatomy, or discrepant readings. This approach is expected to reduce over-instrumentation and minimize unnecessary exposures [7,10-13,15-19,22]. While integrated motor-EALs may appear convenient, findings do not confirm them to be more accurate than dedicated EALs [11,25]. Furthermore, several studies revealed that the use of radiographs alone leads to a tendency to overestimate WL; this supports the use of routine EAL corroboration [10,13-17,19]. Clinicians should also be mindful that radiographic “good length” can still be histologically long or short due to foramen/apex mismatch [3,4,7]. In sum, an EAL-first, radiograph-supported workflow aligns accuracy with prudent imaging.

Strengths and limitations of the findings

The inclusion of RCTs alongside in vivo and ex vivo studies strengthens the reliability of the findings. Nonetheless, there are methodological concerns to be aware of, such as small sample sizes, incomplete reporting of randomization/blinding, absence of protocol registration in most RCTs, spectrum limitations (single-rooted teeth), and inconsistencies in reference standards [11-13,16,20-25]. Moreover, 10 potentially relevant reports were not retrievable despite attempts, and this may have introduced availability bias. Due to these limitations and heterogeneous outcomes, a meta-analysis was not appropriate. Overall, the certainty of evidence is best summarized as moderate.

Recommendations for Future Research

Future studies should include well-powered, prospectively registered RCTs that enroll multirooted/complex teeth and pre-specify ±0.5 mm as the primary accuracy threshold (with ±1.0 mm as the secondary threshold). Studies should also adopt transparent reference standards, including direct microscopic confirmation if feasible, and take into account canal conditions and irrigants. In addition, studies should use identical protocols for a more accurate comparison of stand-alone versus integrated EALs [8,9,11,16,18,23,25]. To further enhance guidance on best clinical practice, other patient-oriented outcomes should also be evaluated, such as repeat-radiograph counts, procedure time, and rates of over/under-fills [10,13,16,19,22].

## Conclusions

Across randomized, in vivo, and ex vivo evidence, electronic apex locators consistently provide more reliable working-length determination than radiographic or tactile methods, translating into fewer clinically relevant errors during preparation and obturation. An EAL-first, radiograph-supported workflow best balances precision with prudent imaging: EALs to target the apical constriction and radiographs reserved for anatomic appraisal, discrepant readings, documentation, and complex cases. Although device-to-device differences appear small, current data do not show endomotor-integrated units to be superior to dedicated EALs. The overall certainty of evidence is limited by spectrum bias toward single-rooted teeth, heterogeneity in reference standards and accuracy thresholds, and modest study sizes, but the direction of effect is stable and clinically meaningful. Routine adoption of EALs is therefore justified in everyday endodontic practice, with future research prioritized toward multirooted and retreatment scenarios, standardized ±0.5 mm primary accuracy thresholds, transparent reference standards, and patient-oriented outcomes such as radiation use, procedure efficiency, and obturation quality.
